# *Pelecocera* (*Pelecocera*) *tricincta* and *Pelecocera* (*Chamaesyrphus*) *caledonica* (Diptera, Syrphidae) reared from *Rhizopogon* fungal host in Finland

**DOI:** 10.3897/BDJ.12.e118563

**Published:** 2024-04-15

**Authors:** Gunilla Ståhls

**Affiliations:** 1 Finnish Museum of Natural History Luomus, Helsinki, Finland Finnish Museum of Natural History Luomus Helsinki Finland

**Keywords:** mycophagy, *Pelecocera*, *Rhizopogonluteolus*, DNA barcoding

## Abstract

MtDNA COI barcodes have frequently been used in identification to associate an unknown life stage in insects with a known species. This study reports the discovery of hoverfly larvae in the fungal fruit bodies of *Rhizopogonluteolus* Fr. & Nordholm, 1817 in Finland. The identity of the larvae was firstly resolved using mtDNA COI barcodes generated from the larvae and tree-based identification confirming the species Pelecocera (Pelecocera) tricincta Meigen, 1822 and Pelecocera (Chamaesyrphus) caledonica (Collin, 1940) (Diptera, Syrphidae). Obtained pupae were reared into adult flies and produced the same two species. The morphological features of these mycophagous larvae are compared with those of other fungus-feeding hoverfly species. This study confirms *Rhizopogonluteolus* as fungal host for these *Pelecocera* species in the Western Palaearctic Region.

## Introduction

*Pelecocera* Meigen, 1822 (Syrphidae, Eristalinae, Rhingiini) is a small genus with eleven species distributed in the Holarctic Region. The molecular phylogenetic study of Rhingiini taxa by Vujić et al. (2018) resolved the genus *Pelecocera* as comprising two subgenera, the monospecific nominal subgenus Pelecocera and the subgenus Chamaesyrphus Mik, 1895 with eight species in the Palaearctic. Nève and Lair (2023) reviewed the classificat history of the genus and its subgenera in the Palaearctic egion. Out of the eight Palaearctic Pelecocerasg.Chamaesyrphus species, all but one occur in geographical Europe (Van Eck and Mengual 2021), as Pelecocera (Chamaesyrphus) japonicus Shiraki, 1956 is only known from Japan. For the Nearctic region, three other Pelecocerasg.Chamaesyrphus species are listed ([Bibr B11048116][Bibr B10976354], Skevington 2020).

Members of the subgenus Chamaesyrphus may be distinguished from *Pelecocera* s.str. by the position and thickness of the antennal arista, which is slender and typically placed dorsobasally on the basoflagellomere in *Chamaesyrphus*, while it is thick and in the apical position in *Pelecocera* s.str.

The feeding mode of the *Pelecocera* spp. larvae has remained unknown and phytophagy has been suggested as the feeding mode (e.g. Speight (2020)). Recently Okada et al. (2021) confirmed mycophagy as the feeding mode of Pelecocera (Chamaesyrphus) japonica. They observed larvae in fungal fruit bodies of *Rhizopogonroseolus* (Corda) Th. Fr., Svensk, 1909 (Blushing False Truffle) and *Rhizopogonluteolus* Fr. & Nordholm, 1817 (Yellow False Truffle) in Japan. They reared some larvae from the fungi and, after the pupation, they obtained adult flies of the species Pelecocera (Chamaesyrphus) japonicus (as *Chamaesyrphusjaponicus)*. In Europe Orengo-Green et al. (2024) provided first morphological description of the immature stages of Pelecocera (Chamaesyrphus) lugubris and Pelecocera (Pelecocera) tricincta reared from *Rhizopogonluteolus* in Denmark.

*Rhizopogon* is a genus of ectomycorrhizal basidiomycetes in the family Rhizopogonaceae (Basidiomycota, Boletales) and it is closely related to the genus *Suillus* (Boletaceae) (Grubisha et al. 2002, Binder and Hibbett 2007). *Rhizopogon* species form hypogeous sporocarps commonly referred to as “false truffles”, but the fruit bodies are of disputed edibility. *Rhizopogon* species are primarily found in ectomycorrhizal association with trees in the family Pinaceae (e.g. Molina and Trappe (2006)). Through their ectomycorrhizal relationships, *Rhizopogon* are thought to play an important role in the ecology of coniferous forests.

MtDNA COI barcodes have frequently been used to associate an unknown life stage in insects with a known species (e.g. Ståhls et al. (2014), Sonet et al. (2013)). In this study, I report the discovery of hoverfly larvae in the fruit bodies (sporocarps) of *Rhizopogonluteolus* Fr. & Nordholm, 1817 in Finland. The species identity of the larvae was initially resolved with mtDNA COI barcodes using tree-based comparison and two hoverfly species were found, namely Pelecocera (Pelecocera) tricincta and Pelecocera (Chamaesyrphus) caledonica (Collin, 1940). The identity of the two hoverfly species was corroborated after rearing the immatures into adult flies and identifying them morphologically as belonging to the same two species.

## Material and methods

### Fieldwork

**Figure 1.**
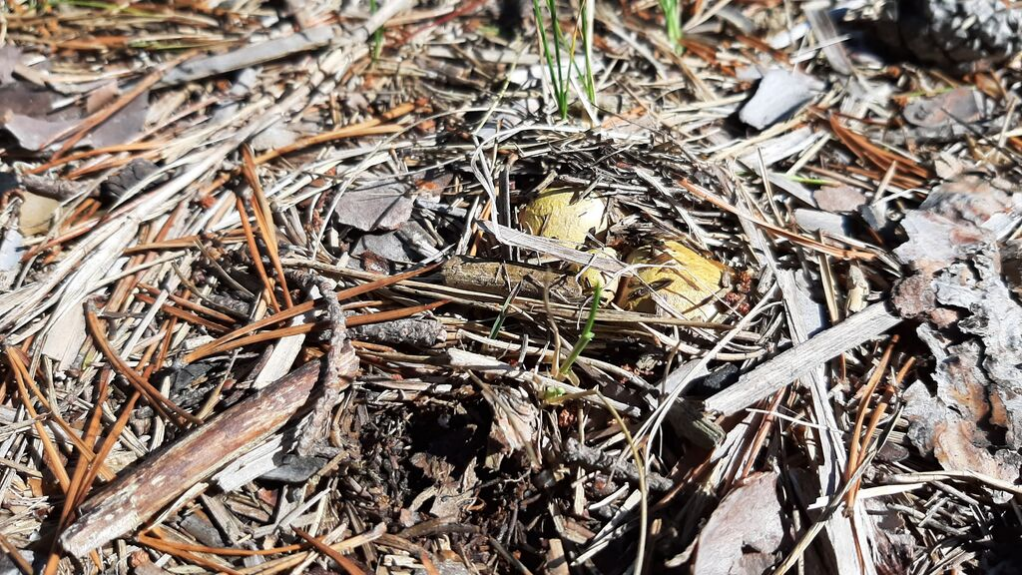
*Rhizopogonluteolus*. Helsinki, Kallahdenniemi. Photo: Gunilla Ståhls.

**Figure 2.**
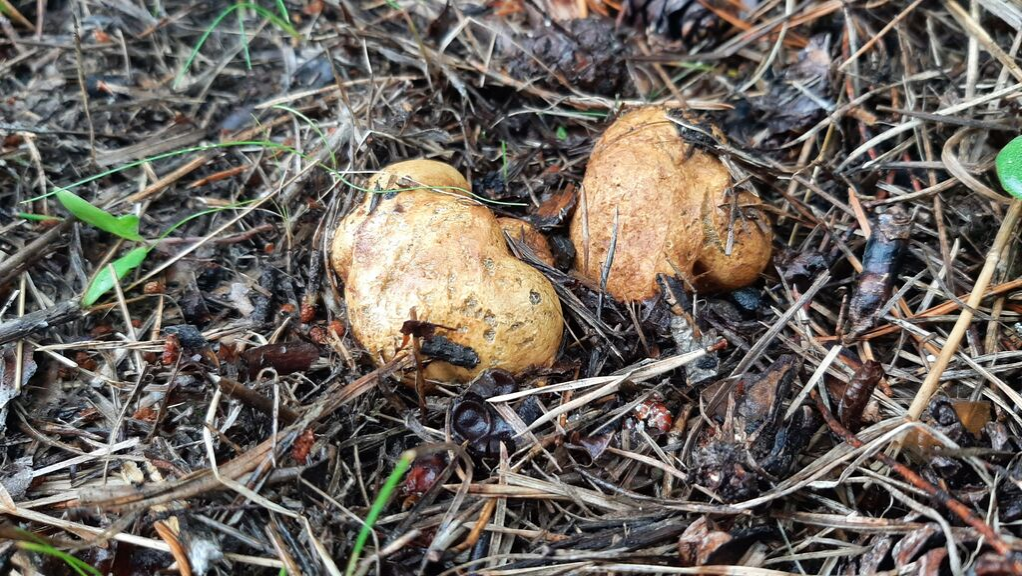
*Rhizopogonluteolus*. Helsinki, Kallahdenniemi. Photo: Gunilla Ståhls.

**Figure 3.**
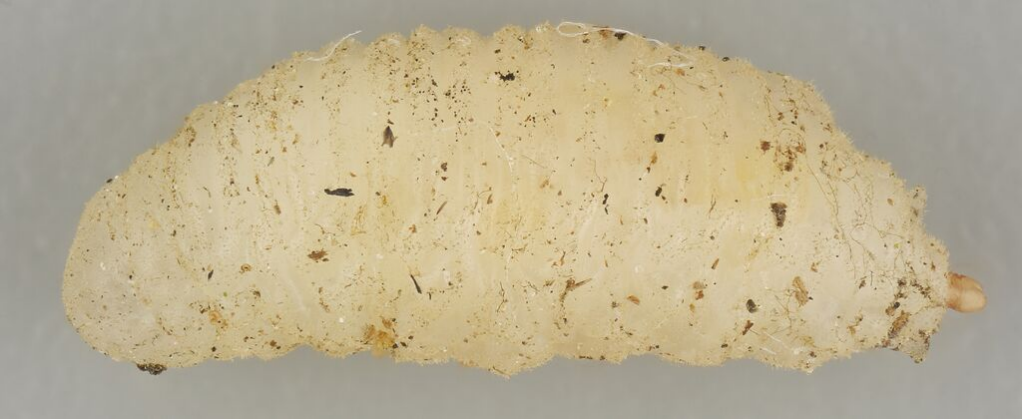
*Pelecocera* spp. larva, lateral view. Photo: Elvira Rättel and Gunilla Ståhls.

The Kallahdenniemi recreational area of the city of Helsinki (60.1870N 25.1445E) is situated in eastern Helsinki (Finland). This sandy soil forested area is dominated by Scots pine (*Pinussylvestris*). From this area, the hoverflies *Pelecoceracaledonica* and *Pelecoceratricincta* have been repeatedly recorded (https://laji.fi/). During several visits to Kallahdenniemi in late August 2023, the mentioned hoverfly species were observed. In autumn 2023, multiple fungal fruit bodies (sporocarps) of *Rhizopogon* sp. were found (Figs 1, 2), occurring in association with both young and mature Scots pines. In the field, some *Rhizopogon* fruit bodies were inspected for the presence of hoverfly larvae by making a slit using a sharp-edged knife to expose the inner fungal tissue for visual inspection. Multiple both young and mature sporocarps of *Rhizopogon* were inspected in Kallahdenniemi and larvae identified as hoverfly larvae were observed in a few sporocarps (Fig. 3). On 17 September and 2 October 2023, altogether six mature sporocarps of brownish-yellow colour (all about 3-4 cm in diameter) were taken for rearing. Sporocarps were placed together in a plastic jar with tissue paper on the bottom and kept outdoors in the shade. The sporocarps decayed into a liquefied olive-brown soup within 2-3 weeks after collection. From the jar with the liquefied sporocarps, five second or third instar larvae were removed and placed individually in tubes for immediate DNA analysis. The plastic jar still contained at least 20 additional hoverfly larvae (smaller and larger) and was kept outdoors until the beginning of November when severe frost nights occurred. Then puparia were searched for in the organic debris (leaves, small twigs etc.) amongst the tissue paper and on the surface area of "the soup". The puparia which had developed pupal horns were removed from the jar and placed individually in Petri dishes for emergence of adult flies. Emerged flies were pinned and the empty puparia glued on cardboard.

### Laboratory procedures

DNA was extracted from each entire larva using the Phire™ Tissue Direct PCR aster Mix #F-170S kit (Thermo Scientific Baltics UAB, Vilnius, Lithuania) following the Dilution & Storage protocol with some modifications. The Phire™ Tissue Direct PCR aster Mix is designed to perform PCR directly from tissue samples with no prior DNA purification. The larva was placed in an Eppendorf tube in 40 µl of Dilution Buffer and 0.8 µl of DNA Release Additive was added. The tube was briefly vortexed and centrifuged and then: 1) incubated at room temperature for about 20 min, 2) placed in +56°C for 10 min and 3) placed in a pre-heated block at 98°C for 2 minutes (after this stage, the larvae were removed and put in individual tubes with about 25% ethanol) and finally centrifuged at 11,000 rpm for 1 min. Two µl of supernatant was used in a 25 µl PCR reaction using the PCR Master Mix solution provided with the kit. The mtDNA COI barcode was PCR-amplified using universal primers LCO1490 and HCO2198 (Folmer et al. 1994) or a shorter fragment using primers Beet (Simon et al. 1994) and HCO2198. Amplified PCR products were electrophoresed on 1.5% agarose gels. Successful amplifications were treated with Exo-SapIT (USB Affymetrix, Ohio, USA) prior to sequencing. The PCR primers were used for sequencing, which was outsourced to the Sequencing Service Laboratory of FIMM Genomics (www.fimm.fi). The sequences were edited for base-calling errors and assembled using Sequencher™ (version 5.0) (Gene Codes Corporation, Ann Arbor, MI, USA) and selected barcode sequences were submitted to GenBank.

### Specimen identification

From the newly-obtained COI barcodes, three full length barcodes were added to a COI barcode data matrix including 25 sequences of *Pelecocera* spp. mined from the NCBI GenBank database (www.ncbi.nih.gov). Tree-based identification (Hebert et al. 2003) was based on comparison of obtained COI barcodes with those available for European species and was with the Neighbor-Joining method (Saitou and Nei 1987) under the Kimura 2-parameter substitution model (Kimura 1980) using the software MEGA11 (Tamura et al. 2021). All positions with less than 95% site coverage were eliminated, i.e. fewer than 5% alignment gaps, missing data and ambiguous bases were allowed at any position (partial deletion option). The barcode sequence of *Pseudopelecoceralatifrons* (Loew, 1856) (GenBank accession number PP446810) was included to root the tree. The identification of the obtained adult flies was confirmed using keys provided in Van Eck and Mengual (2021).

### Digital images

Stacked images of specimen external morphology were taken with a Canon EOS 40S digital camera using d-cell software v. 5.1. Images were combined using Zerene stacker software v. 2, based on 50-100 exposures of the subjects.

## Data resources

Obtained specimens are deposited in the Entomological collections of the Finnish Museum of Natural History (MZH) and registered in the Collections Management System Kotka. The Finnish Museum of Natural History Luomus uses the CETAF stable identifier system, based on http Unique Resource Identifiers (HTTP-URIs) for all collection objects. The specimen data for adults and immatures of both species associated with this study is accessible through the Finnish Biodiversity Information Facility (https://laji.fi/) under the following permanent link: http://tun.fi/HBF.85046?locale=en. New COI barcode sequences of *Pelecocera* spp. were submitted to GenBank under accession numbers OR941126-OR941128 and PP446810 for *Pseudopelecoceralatifrons*.

## Results

### Host fungus

The sporocarps or fruit bodies were readily identified as *Rhizopogonluteolus*. The sporocarps of *Rhizopogonluteolus* are 1.5-5 cm in diameter, without stem, variable in shape being roundish, ovoid or of irregular shape (Grubisha et al. 2002). The fruit bodies are initially yellow in colour (Fig. 1), but turning brownish-yellow upon maturity (Fig. 2). The outer skin is tough and the inner tissue (gleba) is initially pale-yellow and solid, but becomes olive-brown and liquefied in mature and senescent fruit bodies. *Rhizopogonluteolus* occurs throughout most of mainland Europe, but is common only in sandy soil, pine-forested parts of northern Europe. In Finland, the species is recorded up to the 68° latitude and fruit bodies occur mainly in August and September (The Finnish Biodiversity Information Facility, https://laji.fi/observation/list?target=MX.236304).

### DNA identification of larvae

**Figure 4.**
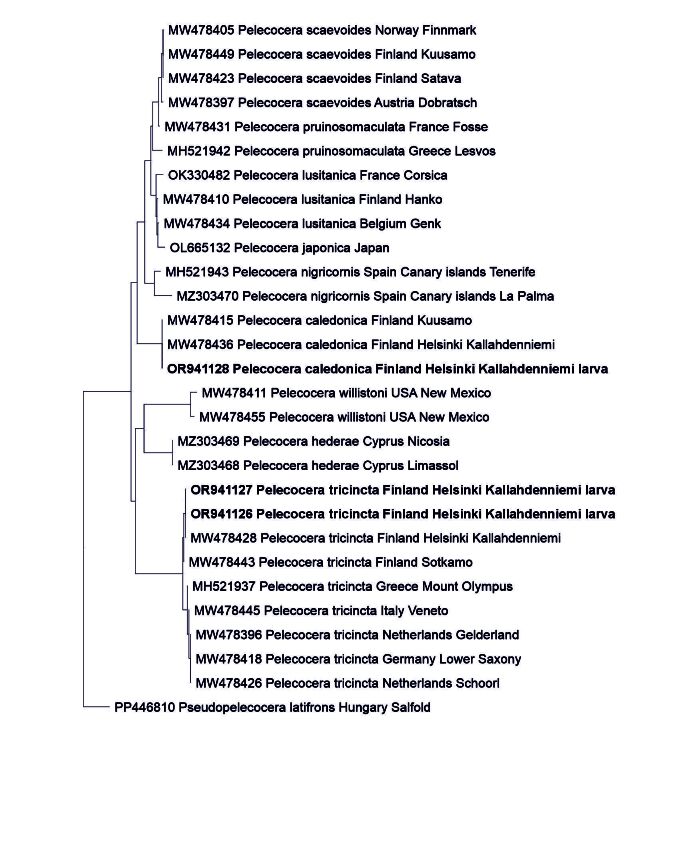
Neighbour-Joining tree for mtDNA COI barcodes of *Pelecocera* spp.

MtDNA COI barcodes obtained from three out of the five tested larvae were used for tree-based identification. The COI barcode dataset used for tree-based identification included altogether 28 nucleotide sequences of *Pelecocera* spp. and *Pseudopelecoceralatifrons* as root of the tree (GenBank accession numbers indicated in Fig. 4). The Neighbour-Joining analysis found the newly-obtained COI barcodes to be identical with those of the species of Pelecocera (Pelecocera) tricincta and Pelecocera (Chamaesyrphus) caledonica, respectively (Fig. 4). The COI barcode sequences for *P.tricincta* comprised 658 nucleotide and for *P.caledonica* 629 nucleotide positions and the DNA sequence matrix comprised 658 positions (inserting ? for the missing data). In the study of Lair et al. (2022), the authors noted that the COI barcodes of *Pelecocerapruinosomaculata* and *P.scaevoides* were resolved as intermixed in their tree, with a mean distance of 0.8% between the taxa. This study utilied part of the same sequences used by Lair et al. (2022) and uses the same taxon names and also recovers the COI barcodes of mentioned taxa as clustering together (Fig. 4).

## Discussion

### Ecology and morphology of *Pelecocera* spp. larvae

Each mature *Rhizopogonluteolus* sporocarp, which was taken from the field for rearing, contained some *Pelecocera* spp. larvae. The rearing process found that the pupae is the overwintering developmental stage. Okada et al. (2021) reported that the *Pelecocerajaponica* larvae they found dwelled in the liquefied decaying inner tissue (gleba) of the specimens of *Rhizopogon* spp. The larvae of *P.tricincta* and *P.caledonica* here reported from *Rhizopogonluteolus* fruit bodies were likewise only found in liquefied fungal tissue of olive-brown colour and with an oily viscosity. The larvae were submerged in the liquefied fungal tissue with their posterior respiratory tube (prp) thrust out of the material. The liquefied material did not fill the inner space of the sporocarrp completely, leaving a non-filled small air space in the dorsal part (about 1/10^th^ of the gleba volume). This agrees with the description of Rotheray (1990) of *Cheilosia* larvae which develop in Boletaceae species. These larvae are also submerged in the decayed Boletaceae fungi of semi-liquid consistenc with the prp out of the decayed material.

**Figure 5.**
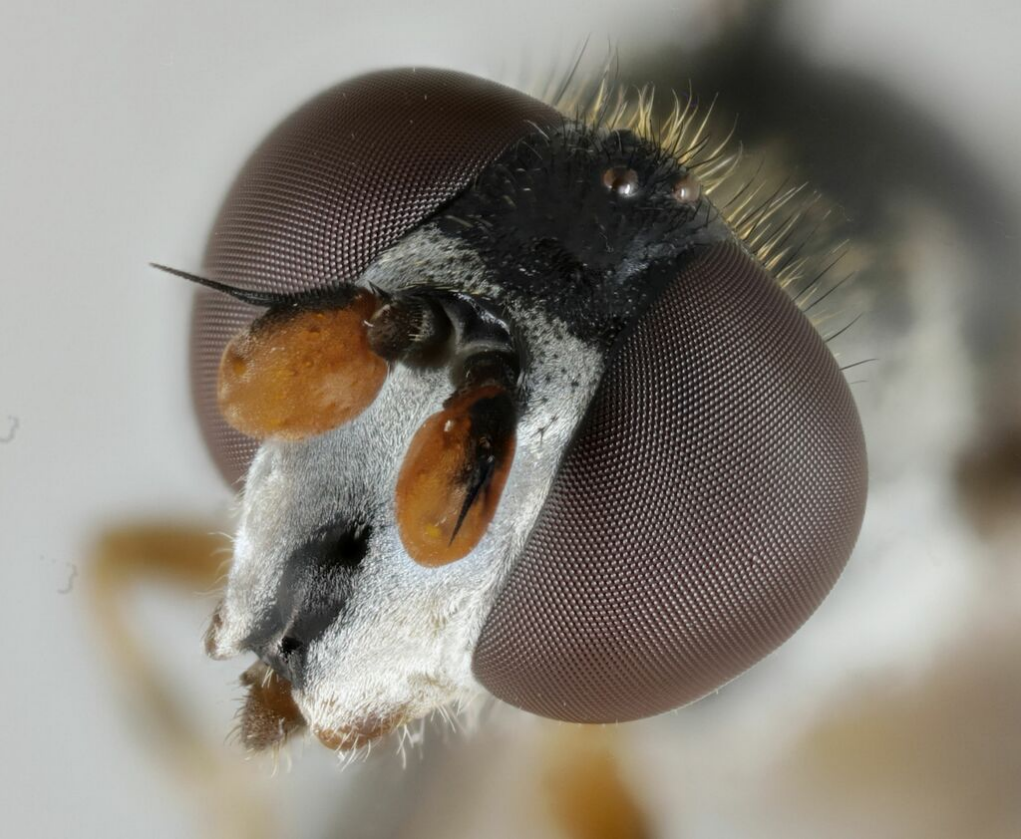
*Pelecoceracaledonica*, female head. Photo: Elvira Rättel and Gunilla Ståhls.

**Figure 6.**
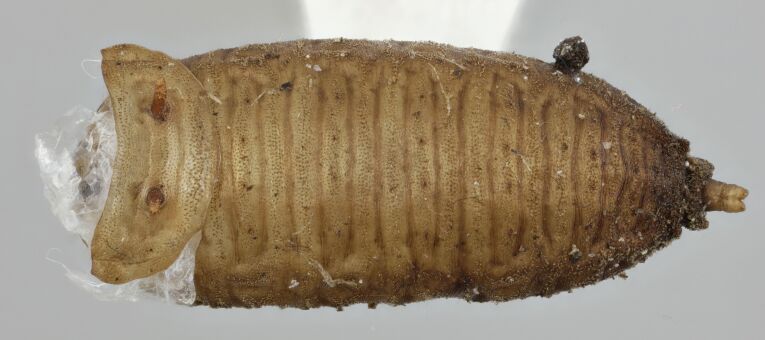
*Pelecoceracaledonica*, empty puparium in dorsal view. Photo: Elvira Rättel and Gunilla Ståhls.

**Figure 7.**
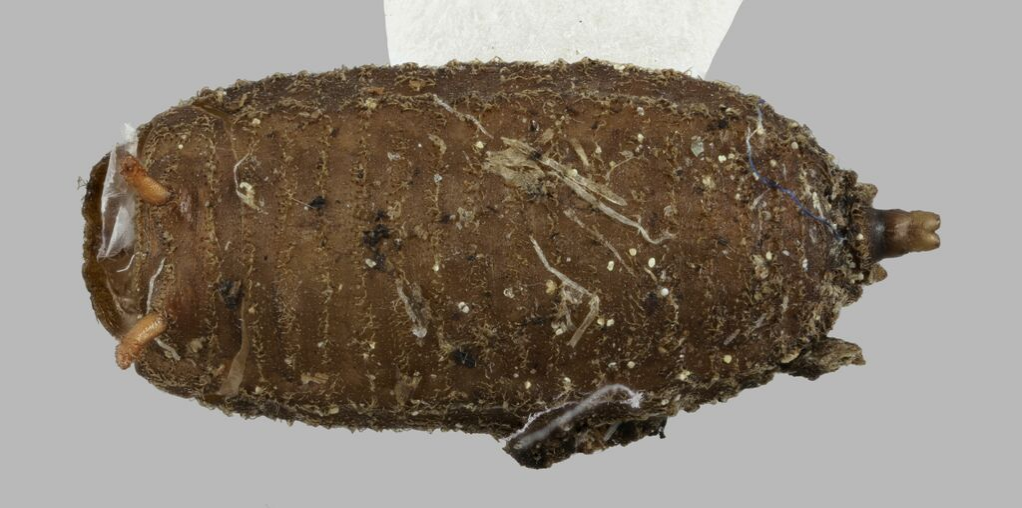
*Pelecoceratricincta*, empty puparium in dorsal view. Photo: Elvira Rättel and Gunilla Ståhls.

**Figure 8.**
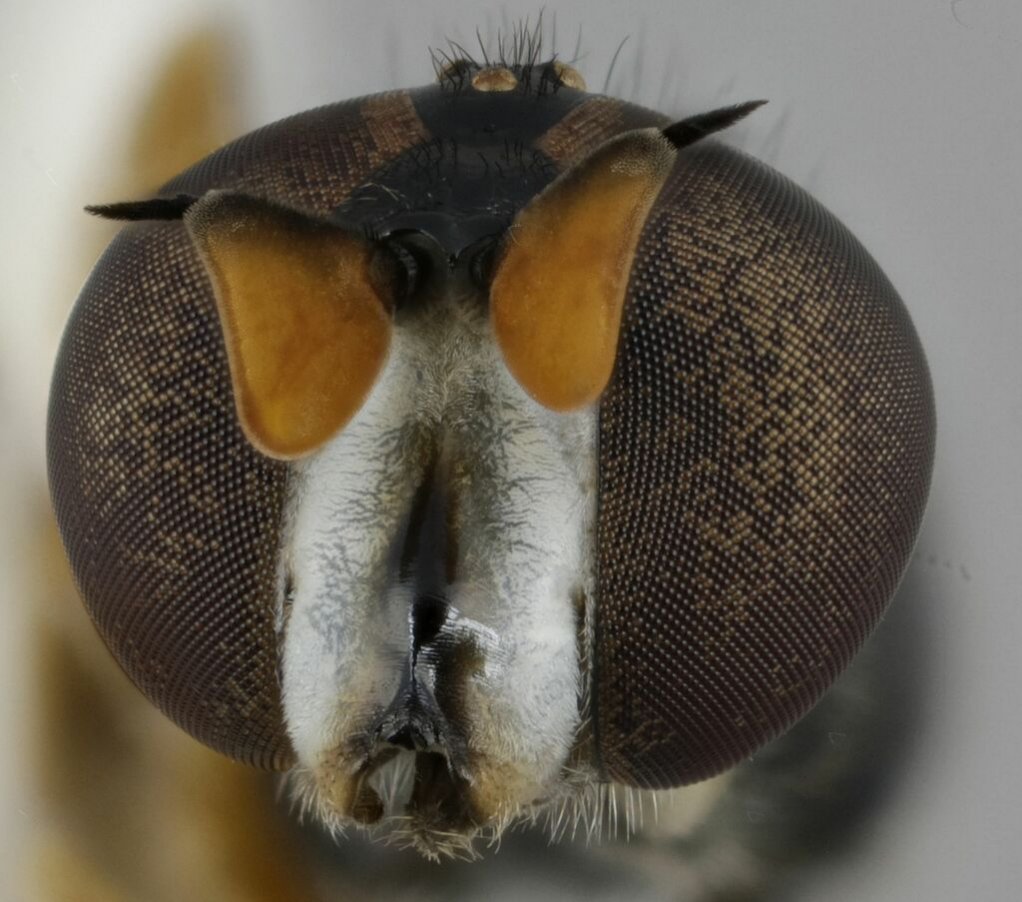
*Pelecoceratricincta*, male head in anterior view. Photo: Elvira Rättel and Gunilla Ståhls.

The mouth parts of the inspected third instar larvae of the *Pelecocera* spp. in this study agree with structures indicated for fungus feeding *Cheilosia* larvae (Rotheray 1990). These structures are a cephalopharyngeal skeleton with a small dorsal mouth-hook not protruding from the mouth and fleshy mandibular lobes and a vestiture of the larvae consisting of rows of setae (Fig. 3). Images are provided for both *Pelecoceratricincta* (Fig. 7) and *Pelecoceracaledonica* to illustrate the vestiture and other structures in the puparia (Fig. 6). Descriptions of the larvae and puparia for both *Pelecoceracaledonica* and *P.tricincta* will be provided elsewhere (in prep.). Photos of the heads of the adult flies of the reared species are provided for morphological identification (Figs 5, 8).

### Conservation aspects

Pennards (2020a) and Pennards (2020b) noted that *Pelecoceracaledonica* and *P.tricincta* are broadly distributed in both northern and southern Europe. His Red List assessment indicated that potential conservation actions for the species should focus on the protection of the habitat where flies occur, but that the species are known to occur in both protected areas and in commercially harvested forests. I searched for False Truffles in Helsinki, Kallahdenniemi in September 2022, with no findings. Comparing monthly rainfalls of year 2022 and 2023, the monthly rainfalls were 2-3x higher for July, August and September in Helsinki in 2023 (Finnish Meteorological Institute https://www.ilmatieteenlaitos.fi/).

In September 2023, the Yellow False Truffles containing *Pelecocera* spp. larvae in the Kallahdenniemi recreational area were found immediately adjacent to footpaths between the public beach and car parking areas and about 10 sporocarps were observed in total in a small area with about 2 m radius. The occurrence of fungi in general is partly dependent on the amount of rainfall during the season, with the 2023 season receiving more rain in southern Finland and many False Truffles were obseved. The observed fruit bodies occurred in (late) autumn when the Kallahdenniemi area is less used by the public. It is not known to which extent human activities (e.g. trampling) could potentially affect the occurrence of the *Rhizopogon* fungi and any immediate conservation actions does, therefore, not seem warranted.

## Conclusions

The findings of at least one specific host fungus species for Pelecocera (Chamaesyrphus) caledonica and Pelecocera (P.) tricincta from northern Europe provide important information for the understanding of the ecology and conservation of the hoverfly species in question. Other *Rhizopogon* spp. sporocarps should be inspected for *Pelecocera* spp. larvae for a more detailed picture of host fungus preferences for all *Pelecocera* species in Europe.
